# The year in review: progress in brain barriers and brain fluid research in 2018

**DOI:** 10.1186/s12987-019-0124-y

**Published:** 2019-02-05

**Authors:** Richard F. Keep, Hazel C. Jones, Lester R. Drewes

**Affiliations:** 1 Department of Neurosurgery, University of Michigan, R5018 BSRB, 109 Zina Pitcher Place, Ann Arbor, MI 48109-2200 USA; 2 Gagle Brook House, Chesterton, Bicester, OX26 1UF UK; 3 Department of Biomedical Sciences, University of Minnesota Medical School Duluth, Duluth, MN 55812 USA

## Abstract

This editorial focuses on the progress made in brain barrier and brain fluid research in 2018. It highlights some recent advances in knowledge and techniques, as well as prevalent themes and controversies. Areas covered include: modeling, the brain endothelium, the neurovascular unit, the blood–CSF barrier and CSF, drug delivery, fluid movement within the brain, the impact of disease states, and heterogeneity.

## Editorial

There continues to be much interest in brain barrier and brain fluid research. Many important papers (too many to be cited here) have been published in the field in 2018. The purpose of this editorial is to highlight some recent advances and themes for the readership of *Fluids and Barriers of the CNS*. As always, the selection of papers discussed is idiosyncratic and the discussions are necessarily brief. The journal welcomes more in-depth reviews as well as novel research articles in any of the areas discussed.

## Modeling

### In vitro blood–brain barrier (BBB)/neurovascular unit (NVU) modeling

Engineering the BBB/NVU in vitro is challenging: models differ in complexity and their ability to replicate different in vivo parameters. The utility of such models varies with the specific questions being addressed. However, there is a question as to what basic parameters such models should exhibit. DeStefano et al. have recently proposed a series of benchmarks for in vitro BBB models [1] that might form a basis for such decisions or lead to a discussion of validation parameters.

One of the recent advances in BBB modeling is the use of human-induced pluripotent stem cells (iPSCs) to derive different cells of the neurovascular unit (e.g. endothelial cells, pericytes, astrocytes). Such models are now being pursued by multiple groups (e.g. [2–7]). The use of human iPSCs may aid in the translation of basic science to the clinic. In addition, it is possible to generate such models from patient-derived iPSCs allowing examination of the impact of patient-specific mutations [8]. Thus, Lee et al. [5] found that brain endothelial cells derived from iPSCs from patients with childhood cerebral adrenoleukodystrophy have impaired barrier properties and accumulate lipid droplets, effects that were rescued by treatment with a block copolymer.

While there are many studies that have examined the role of different cell types (particularly endothelial cells, astrocytes and pericytes) on the characteristics of in vitro NVU/BBB models, the importance of extracellular matrix has generally received less attention. Katt et al. [4] and Al-Ahmad et al. [2] recently showed the importance of different extracellular matrix molecules on iPSC-derived BBB models. BBB modeling is a fast developing field (reviewed in [9]). One of the goals of multiple groups is the development of a ‘barrier-on-a-chip’. Such chips may be very useful for drug testing and alternative designs are reported [10–12].

### Computer modeling

As well in vitro modeling, there have been a number of important studies using computer (in silico) modeling in 2018, particularly in relation to the movement of fluid through the proposed glymphatic system. Thus, Faghih et al. [13] and Rey and Sarntinoranont [14] modeled fluid movement within the perivascular space. Their findings throw skepticism on the importance of perivascular bulk fluid flow, an essential component of the glymphatic system. Another example of in silico modeling are efforts to predict brain:blood unbound concentration ratios for different drugs and, thus, reduce the expense of developing brain therapies [15, 16].

### Animal modeling

The use of non-mammalian models to examine the blood–brain, blood–retinal and blood–CSF barriers continues to gain interest [17]. The relative ease and lower cost of genetic manipulation and brain visualization has spurred the use of zebrafish for barrier studies [18, 19], while others have started examining relevant barriers in drosophila (fruit flies). Thus, Zhang et al. [20] examined the perineural glia of drosophila that form the insect equivalent of a ‘blood’-brain barrier and showed a circadian rhythm in barrier function.

## Brain endothelium

### Brain endothelial junctions: importance, structure and regulation

Brain endothelial tight junctions (TJs) are an essential component of the NVU/BBB, helping protect the brain from potentially harmful blood-borne compounds. TJ disruption can contribute to brain injury in a number of types of brain injury or disease. In 2017, Menard et al. [21] found that social stress in mice, a model of depression, reduced expression of claudin-5, that reducing claudin-5 experimentally could induce depression-like symptoms and that treatment with a known anti-depressant increased claudin-5 expression. These results suggest an important impact of changes in the NVU/BBB on brain function and animal behavior. Cheng et al. [22] recently examined a prolonged learned ‘helplessness’ model of depression in rats and found that BBB disruption contributed to an inability to recover from the imposed stress. Another study examining the basis of psychiatric disease (schizophrenia) suggests a link between neural cell and cerebrovascular development [23]. Furthermore, Todd et al. [24] recently used focused ultrasound to locally ‘open’ the BBB in rats and found this treatment disrupted both local and inter-hemispheric functional connectivity within the brain, again indicating an important role of NVU/BBB integrity in overall brain function. While adoption of focused ultrasound is progressing for enhancing brain drug delivery, safety concerns for this technology also need to be addressed [25, 26], including subtle behavioral effects. Overall, the link between the cerebrovasculature and behavior is greatly understudied.

There have been a number of important studies in 2018 on the regulation of brain endothelial TJs in health and disease with some focusing on cell signaling pathways. For example, Isawa et al. [27] showed the importance of β1-integrin and extracellular matrix interactions in regulating endothelial tight junctions. Other recent studies documented the importance of lysophosphatidic acid, Wnt, PI3K/Akt and tumor necrosis factor-α signaling in regulating brain endothelial TJs [28, 29]. Wang et al. [30] identified microRNA-130a as an important regulator of BBB function in cerebral ischemia and observed that this microRNA downregulates occludin expression by inhibiting Homebox A5 expression. That study is one of several demonstrating the importance of microRNAs in up- or down-regulating brain endothelial permeability (e.g. microRNA-143, -146a, -149-5p, -21, -96 and -155 [31–36]). Interestingly, in addition, Ma et al. [37] found that claudin-5 affects the expression of long non-coding RNAs (lncRNAs) in brain endothelial cells thereby regulating BBB properties in brain metastases.

Most current work on brain endothelial TJ regulation employs animals or animal-derived cells. There is a concern that species differences, e.g. in humans, in regulation may exist. Wang et al. [38] examined the potential role of one protein, periaxin, present in brain endothelial cells in humans but not in multiple other species. They found that periaxin strengthens barrier permeability and attenuates the expression of inflammatory mediators.

In terms of brain endothelial junction proteins, most attention has traditionally focused on the role of TJ proteins, although the importance of adherens junction proteins in barrier formation and function is recognized [39]. A recent study highlights the importance of brain endothelial gap junctions, and particularly connexin-43, in the barrier changes that occur in cerebral cavernous malformations type III by remodeling the TJs [40].

There is much interest in developing methods to reduce or increase TJ protein expression in brain endothelial cells to either enhance drug delivery or improve barrier function (e.g. in disease). One approach has been the use of claudin-targeted peptides that induce claudin internalization/degradation. Sladojevic et al. [41] reported that cerebral ischemia induces the novel expression of claudin-1 in the cerebral endothelium and that reducing its expression, with a claudin-1 targeted peptide, improves endothelial barrier permeability long-term.

### Brain endothelial transcellular transport

Brain endothelial cells possess a wide range of transporters that are important in facilitating the entry of compounds into brain (such as the glucose transporter, GLUT1) or preventing their entry (such as multidrug resistance protein 1, P-glycoprotein). One difficulty in assessing the importance of different transporters in nutrient or xenobiotic deposition is that they often have overlapping substrate specificities and the use of quantitative proteomics to assess transporter expression continues to grow. An example is a recent study by Al-Feteisi et al. [42] who reported on rat brain microvessel transporters. This approach can also be used to compare transporter expression across different barrier tissues, such as the arachnoid-epithelial cell barrier [43].

The relative importance of BBB-mediated efflux of different compounds from brain parenchyma versus alternative egress routes (particularly the perivascular pathway) is the subject of a major review by Hladky and Barrand [44]. This is a very important and controversial topic impacting normal brain function, multiple disease states (e.g. β-amyloid clearance in Alzheimer’s disease) and drug delivery.

Because brain to blood efflux (e.g. via ATP-biding cassette (ABC) transporters) has an important role in regulating both the brain concentrations of endogenous compounds and xenobiotics, there is great interest in how such transport is regulated. Recent examples include reports by Hartz et al. [45], who found that preventing P-glycoprotein ubiquitination could be used to decrease β-amyloid levels in a mouse model of Alzheimer’s disease; Xie et al. [46] who provide evidence that microRNA-298 regulates P-glycoprotein; and Shin et al. [47] who showed that estrogen represses breast cancer resistance protein expression and activity in brain endothelial cells after ischemia. One neglected aspect of blood–brain transport studies is whether there may be sex differences. Brzica et al. [48] recently found that there are differences in adult male and female rats in the expression and activity of organic anion transporting polypeptide 1a4 in rat brain microvessels. Whether similar differences exist in humans is an important question because of the implications for drug delivery.

Another transcellular route across the cerebral endothelium is vesicular trafficking. This is particularly important for proteins, including potentially therapeutic antibodies. A better understanding of trafficking mechanisms and vesicle fate (e.g. transcytosis vs lysosomal degradation) will aid in such therapeutic studies. Haqqani et al. [49] recently delineated antibody trafficking between different endosomal compartments. Such mechanisms may differ between species and Ribecco-Lutkiewicz et al. [6] recently found that a human iPSC-derived BBB model can be used to study receptor-mediated transcytosis triggered by antibodies. An important question is how brain endothelial transcytosis may be impacted by particular disease states? Sadeghian et al. [50] recently found that cortical spreading depolarizations triggered caveolin-1 dependent endothelial transcytosis.

One important aspect of blood–brain transcellular transport mechanisms is that they may be ‘hijacked’ by certain viruses allowing brain penetration. The recent Brazilian Zika virus outbreak caused microcephaly and other neurological conditions. Alimonti et al. [3] used an in vitro BBB model derived from human iPSCs to examine mechanisms of virus transport. They found evidence for transcellular penetration involving a receptor tyrosine kinase, AXL, suggesting potential targets for therapeutic interventions.

## Neurovascular unit

### Neurovascular signaling

The cells of the NVU (including, endothelial cells, astrocytes, pericytes neurons and perivascular macrophages) play an important role in regulating cerebrovascular function, including permeability [51]. Most attention has focused on the role of secreted peptides/proteins (e.g. [52]) and, more recently, lipids such as sphingolipids [53], by astrocytes and pericytes in such regulation. However, the importance of other signaling mechanisms is becoming clear. This includes a role of extracellular vesicles/exosomes (reviewed in [54]) and microRNAs (see above). Extracellular vesicles are released by a wide range of cell types and can contain a variety of signaling molecules including proteins, microRNAs and mRNAs. Another intriguing possible signaling mechanism has been suggested by Errede et al. [55] who described pericyte-derived tunneling nanotubes that may be involved in pericyte to endothelial cell signaling during normal and pathological angiogenesis.

While much attention has focused on the impact of pericyte-, astrocyte- and neuronal-derived signals on the brain endothelium, there are also important endothelial-derived signals that affect parenchymal cells. An example of such pathways was recently described by Segarra et al. [56] who found that endothelial disabled1 (Dab1) is an important regulator within the NVU. Deletion of endothelial Dab1 reduces laminin-α4 secretion and thereby decreases integrin-β1 signaling in astrocytes that, in turn, regulates both neuronal migration and BBB function.

### Endothelial glycocalyx and the vascular basement membrane

Two components of the NVU that receive relatively little attention are the endothelial glycocalyx and the vascular basement membrane. Kutuzov et al. [57] examined the penetration of different sized tracers into brain and found that the glycocalyx on the luminal membrane of the cerebral endothelium is a significant barrier to the penetration of large molecular weight dextrans. There is also recent evidence that preserving the glycocalyx after cardiac arrest in rats helps preserve barrier function and reduce brain edema formation [58].

As noted above, in vitro evidence indicates the importance of extracellular matrix components in determining vascular wall properties [4]. The neurovascular basement membrane is secreted by multiple cell types (e.g. endothelial cells, pericytes and astrocytes). The molecular complexity of the vascular basement membrane and its structural compartments within the vascular bed are now delineated by Hannocks et al. [59] using cellular and molecular markers. This group of investigators propose that the capillary endothelial cell- and perivascular cell-derived proteins are physically separated, potentially providing a route for perivascular fluid flow [59, 60] (see below).

## The blood-CSF barrier and CSF

### Choroid plexus and CSF

The role of the choroid plexus in neuroinflammation continues to garner interest, including mechanisms for combatting infection [61, 62]. Interestingly, the choroid plexus produces high levels of the ‘anti-aging’ protein klotho and recent evidence indicates that klotho has a major role in regulating the brain/immune interface at the choroid plexus [63]. Other results have shown that the choroid plexus forms an important niche for T cell activation within the brain [64].

Progress continues to be made on identifying ion transporters at the choroid plexus and determining their role in regulating fluid movement and CSF composition [65–67]. For example, Preston et al. [68] examined the role of the transient receptor potential vanilloid 4 (TRPV4) cation channel at the choroid plexus epithelium and report that activating this channel caused an immediate increase in transepithelial ion flux. Modulating this channel may be a novel way of controlling choroid plexus ion transport and, potentially, CSF secretion. Another notable finding is that a significant portion of CSF production may result from molecular transfer of water via the action of a Na/K/2Cl cotransporter (NKCC1) expressed on the CSF-facing membrane of the choroid plexus [69]. This novel mechanism, in concert with aquaporins, may be responsible for nearly half of the water flux during CSF production.

Several neurological conditions result in changes in CSF composition and, therefore, CSF is widely used in disease biomarker studies. For example, the use of CSF biomarkers in Alzheimer’s disease, cerebral amyloid angiopathy and other neurodegenerative diseases, is emerging, although there are still areas of technical and conceptual controversy (see reviews in [70–73]). While the use of blood-borne biomarkers may have major advantages, there are still hurdles to overcome for that approach [74].

Changes in choroid plexus function with disease may contribute to changes in CSF composition. Stopa et al. [75] examined changes in the choroid plexus transcriptome in patients with neurodegenerative disorders (Alzheimer’s disease, frontotemporal dementia and Huntington’s disease) and found both common and disease-specific changes. These might be involved in brain damage but also brain protection and repair.

While most focus has been on CSF proteins as biomarkers, there are starting to be growing numbers of studies examining RNA and DNA in CSF. For example, Dos Santos et al. found evidence that a panel of microRNAs in CSF could be used to detect early Parkinson’s disease [76]. There has also been interest in using CSF DNA to examine the presence of mutations or transcript copy number variations associated with brain tumors [77].

### Arachnoid membrane

Compared to the NVU/BBB and the blood-CSF barrier at the choroid plexus, the blood-CSF barrier at the arachnoid membrane has received very little attention. Zhang et al. [43] recently used quantitative proteomics to compare transporter expression in the leptomeninges vs. choroid plexus in rat. While some transporters were enriched in choroid plexus, others were much more highly expressed in the leptomeninges including multidrug resistance protein 1 (p-glycoprotein), breast cancer resistance protein (bcrp) and organic anion transporter-1. The role of the arachnoid membrane in brain homeostasis and drug disposition deserves greater attention.

One potential reason for the high expression of transporters in the leptomeninges is suggested by the recent finding that the leptomeninges are the primary source of prostaglandin D2, which is involved in sleep regulation [78]. This adds to a growing literature indicating that the leptomeninges are a source of neuroactive/neurotropic factors. Transporter expression at the leptomeninges may be involved in regulating the concentration and distribution of such factors.

## Drug delivery

With the rising capabilities of immunotherapy, there is great interest in the delivery of therapeutic antibodies to the brain for a variety of neurological conditions, including Alzheimer’s disease and advances are being made in the design of antibodies to enhance CNS delivery (reviewed by Stanimirovic et al. [79]). Most preclinical work on antibody delivery is performed in rodents and differences between species are a concern for translational applications. Wang et al. [80] examined whether the CSF/serum ratio after systemic antibody delivery differs between rats and cynomolgus monkeys and found good agreement between those two species.

Alternative approaches to delivering antibodies to the brain involve bypassing the BBB via either the intranasal route or direct CNS (usually intrathecal) administration. The utility of such approaches in accessing particular brain areas or cell types requires knowledge of how antibodies move within the CNS. Pizzo et al. [60] examined how IgG and smaller single-domain antibodies move within the rat brain after intrathecal administration, with a particular focus on perivascular transport. The perivascular route allowed the penetration of these macromolecules deep within the brain in contrast to diffusion across the brain surface. Interestingly, they found evidence of perivascular transport of these molecules in all vessels, including capillaries (see below) and reported that perivascular flow was enhanced by osmolyte co-infusion with the antibodies.

Much of the in vivo evidence on small drug penetration into brain has been derived from rodent studies. There are concerns that there may be differences between species in drug penetration, particularly if there is a transporter component in either influx or efflux. There has, therefore, been interest in developing humanized mouse models expressing human transporters. Sano et al. [81] reports development of such a mouse expressing the organic anion transporter OATP1A2 in the brain endothelial cell although, unfortunately, it had little effect on the brain penetration of the substrates examined.

There continues to be great interest in developing methods to disrupt the BBB to enhance drug delivery. One approach is the use of focused ultrasound and Arvantis et al. [82] reports studies on a mouse model of brain metastases. Interestingly, they found that focused ultrasound in combination with microbubbles not only enhanced barrier permeability, it also increased interstitial convective transport.

A proposed alternate approach for increasing drug delivery via enhanced neurovascular permeability is to use endogenous signaling pathways that regulate barrier function. There is preclinical evidence that activation of the adenosine A2A receptor can cause transient enhanced permeability and Jackson et al. [83] examined whether an A2A receptor agonist, regadenoson, could enhance temozolomide concentrations in brain in glioblastoma patients. The initial results indicated no enhancement. This again shows the difficulty of translating preclinical data to patients and further pharmacodynamic and pharmacokinetic studies are needed.

## Fluid movement within the brain

*Fluids and Barriers of the CNS* is currently producing a thematic series on this subject, entitled, *CNS Fluid and Solute Movement: Physiology, Modeling and Imaging*.

### Perivascular and parenchymal fluid flow

The concept of a glymphatic system has engendered a surge in interest in fluid (and associated solute) flow within the brain [84]. The proposed glymphatic system involves fluid entry into brain along the arterial perivascular space, fluid movement through brain parenchyma that is astrocyte and aquaporin-4 dependent, and fluid exit from brain along the venous perivascular space. There is evidence that this system is altered by variables such as exercise [85], circadian rhythm [86] and disease states [84]. However, recently the concept of a glymphatic system has been vigorously debated (for reviews see [84, 87]).

Most evidence for the glymphatic system comes from studies on perivascular flow using two-photon microscopy and also now with Magnetic Resonance Imaging [88–90]. However, it should be noted that even that component has been questioned. For example, Faghih et al. [13] have tried to computer model fluid flow within the glymphatic system and found it implausible based on current anatomical and pressure gradient data. Another modeling paper, Rey and Sarntinoranont [14], also predicted that fluid flow in the perivascular space would be oscillatory with no net flow over time. Most studies of the glymphatic system are currently based on solute tracking rather than measuring fluid flow and new techniques to examine the latter could be very informative.

The parenchymal component (astrocyte/aquaporin-4 mediated) of the glymphatic system has been the most difficult to study. However, Huber et al. [91] did recently report that an aquaporin-4 facilitator promotes brain interstitial fluid circulation. Recently, a potential alternate link between the periarterial and perivenous spaces has been proposed for fluid and solute flow, a pericapillary space [60]. Anatomically, the vascular basement membrane is secreted by endothelial cells and perivascular cells (pericytes/astrocytes) and a gap between these types of the basement membrane may form the basis of a pericapillary space [59]. Also in contrast to the original glymphatic hypothesis, a recent study by Albargothy et al. [92] concluded that tracers in the CSF pass into the brain parenchyma along the pia-glial basement membrane alongside arteries and exit the brain along intramural pericapillary and periarterial basement membranes.

One proposed role of the glymphatic system is in the clearance of potentially toxic peptides/proteins from the brain, including β-amyloid [84]. The evidence on the relative importance of perivascular drainage of β-amyloid versus BBB transport has recently been reviewed in depth by Hladky and Barrand [44]. They contend that the current evidence favors the BBB as being the most important route.

### CSF flow and drainage

CSF flow and the role of the choroid plexus continues to be somewhat of a contentious subject [93]. However, recent evidence shows the critical importance of CSF flow. Petrik et al. [94] found that alterations in ventricular fluid flow promote proliferation in subependymal zone neural stem cells in mice by eliciting Na and Ca signaling in those cells. This signaling only occurred in neural stem cells in contact with ventricular fluid. Thus, CSF flow has a central role in regulating adult neurogenesis and the implications of this for conditions with altered CSF flow, e.g. hydrocephalus, need to be explored.

The classical view that CSF drainage occurs at the arachnoid villi of the sagittal and transverse sinuses has been challenged for decades with a growing understanding of the importance of drainage across the cribriform plate to the nasal lymphatics/cervical lymph nodes and drainage via the spinal nerve roots to the lumbar lymph. More recently, the importance of meningeal lymphatics that also drain to the cervical lymph nodes has been highlighted. Da Mesquita et al. [95] examined the effects of manipulating meningeal lymphatics using a variety of techniques to inhibit drainage in mice and vascular endothelial growth factor C to enhance those lymphatics. They found that meningeal lymphatic dysfunction aggravates age-associated cognitive decline, Alzheimer’s disease pathology and β-amyloid accumulation, while enhancing lymphatic drainage improved learning and memory in mice. Similarly, Louveau et al. [96] provides evidence on the importance of meningeal lymphatics in neuroinflammation. Ma et al. recently found using fluorescent tracers and imaging in awake and anesthetized mice, that tracers exited to the lymphatic system faster with slower spread into the brain perivascular spaces in awake mice than when anesthetized [97].

## Disease states

### Hydrocephalus

Congenital hydrocephalus is difficult to treat successfully and has a variety of causes. Fetal MRI can reveal anatomic features that predict aqueduct stenosis which can help in subsequent obstetric management [98]. There continues to be an interest in the role of ependymal cilia in the development of hydrocephalus. Abdelhamed et al. [99] found that a homozygous splice site mutation in the coiled-coil domain containing 39 (Ccdc39) protein causes the progressive hydrocephalus that occurs in the prh mouse. Those mice develop shorter ependymal cilia with disorganized microtubules. Similarly, mouse models lacking the ciliary proteins CFAP221, CFAP54 and SPEF2 all develop hydrocephalus, the severity of which depends on the background strain. McKenzie et al. [100] have examined the genetic basis of that variation, identifying genes involved in brain and cilia development and function. Malfunctioning genes that regulate neural tube development and neural stem cell fate also lead to abnormal neurogenesis and congenital hydrocephalus [101].

In premature infants and in adults, post-hemorrhagic hydrocephalus is a major clinical problem and the Hydrocephalus Association hosted a conference on the topic to discuss opportunities for research and encourage further research efforts. A summary of that meeting has been published [102]. There has long been an interest in whether choroid plexus ion transport might be targeted as a therapy for hydrocephalus. For example, in 2017 Karimy et al. [103] provided preclinical data that intraventricular hemorrhage causes CSF hypersecretion by stimulating choroid plexus Na/K/Cl cotransport. In 2018, Li et al. [104] found that germinal matrix hemorrhage in neonatal rats induces upregulation of the sodium-coupled bicarbonate exchanger (NCBE) and targeting that transporter using small interfering RNAs reduces post-hemorrhagic hydrocephalus.

Idiopathic intracranial hypertension is a syndrome of unknown cause in obese patients with high intracranial pressure, without ventriculomegaly but with associated visual disturbances and morbidity. A multivariate analysis showed that anemia and NSAID use were risk factors [105]. There is some evidence that the condition is related to abnormal pressure in the cranial venous sinuses affecting CSF drainage [106, 107].

The pathogenesis of normal pressure hydrocephalus (NPH), which occurs in the elderly, is unclear. A large group of possible NPH patients studied in Finland showed that confirmed NPH patients had a higher incidence of hypertension or type-2 diabetes mellitus and that cardiovascular and cerebrovascular disease was the most frequent cause of death [108]. A cohort of Finish and Norwegian NPH patients was found to have a fourfold higher incidence of copy number loss in intron 2 of *SFMBT1* than normal although the pathogenic role was not clear [109]. Treatment is usually performed when patients show classic symptoms of gait disturbance, mental and urinary problems. A small study however, has shown that patients develop enlarged ventricles at least 3 years before symptoms are apparent, suggesting possible implications for management [110]. Diagnosis most often relies on invasive infusion techniques. For example, as part of a clinical trial, the intracranial CSF dynamic profile was studied by infusion in NPH patients and found to differ from that of healthy controls [111]. Recently, an MRI study on healthy individuals by Burman et al. [112] determined the relative distribution of compliance between the spinal and cranial CSF compartments and proposed a model that can be used to estimate both the cranial compliance and intracranial pressure non-invasively. Hence, non-invasive MRI techniques continue to be a promising route to aid diagnosis and determine shunt surgery response. Computerized volumetric analysis of the CSF spaces enabled good discrimination between NPH and brain atrophy patients [113] and the apparent diffusion coefficient of water measured in different brain regions can distinguish between NPH patients and patients with vascular dementia or Alzheimer’s disease [114]. On the other hand, specific features of MR images in NPH patients are not good predictors for reversibility of symptoms after shunt surgery [115]. The same group reported that around 40% show improved symptoms after surgery [116]. Many CSF biomarkers continue to be investigated as a tool to predict treatment outcome for NPH: promising compounds suggested to warrant further investigation are Aβ42, tau, p-tau, neurofilament light protein (NFL) and leucine-rich α-2-glycoprotein (LRG) [117].

### Role of NVU/BBB changes in neurological conditions

NVU/BBB functions are altered in many neurological conditions. There have been multiple reviews in 2018 outlining the current state of knowledge with regards to Alzheimer’s disease and other neurodegenerative diseases [118–120], ischemic stroke [51, 121], hemorrhagic stroke [122], multiple sclerosis [51] and primary and metastatic brain cancer [51]. Importantly, these reviews serve to highlight that NVU/BBB changes are not just a consequence of parenchymal injury, but may actually contribute to that injury and are a therapeutic target. Thus, for example, targeting the vascular changes in Alzheimer’s disease and cerebral ischemia may reduce disease progression [120, 121]. Recent results also indicate that brain endothelial cell dysfunction is the underlying cause of white matter injury in cerebral small vessel disease [123]. That is not to say that parenchymal cell dysfunction may not cause NVU/BBB dysfunction. For example, Rempe et al. [124] recently showed that neuronal glutamate release in epilepsy causes matrix metalloproteinase-2 and -9 upregulation that, in turn, causes BBB disruption and may further impact the brain.

The mechanisms by which NVU/BBB dysfunction may cause parenchymal cell injury in different neurological conditions are under investigation. There are multiple neuroactive compounds present in plasma that may gain entry into brain and participate in injury. One extensively studied compound is fibrinogen that has pleiotropic roles in CNS inflammation [125].

Another interesting question is what severity of brain injury is required to cause NVU/BBB dysfunction. A recent porcine study suggests that concussion causes mechanical BBB disruption [126]. Indeed, a study on high school American football players indicates that even sub-concussive (clinically asymptomatic) high acceleration hits during a season result in elevated levels of brain injury markers in serum [127]. The appearance of these brain proteins (tau and ubiquitin C-terminal hydrolase L1) in serum may involve some NVU/BBB disruption. Tagge et al. [128], studying mice also found that closed-head injuries can cause neuropathology including microvascular injury independent of signs of concussion.

## Heterogeneity

### Temporal heterogeneity

Far from being static entities, the blood–brain barriers show dynamic change with time (long- and short-term). While there have been many studies examining the development of the blood–brain and blood-CSF barriers, including in 2018 (e.g. [19, 129]), the impact of ageing on these barriers is still receiving relative little attention. Such changes may participate in disease susceptibility, even in people without neurodegenerative diseases. Goodall et al. [130] examined the impact of ageing in humans and mice on BBB function. They found increased TJ breaks in mice with age and increased brain vascular permeability (protein extravasation) in humans with age. Similarly, Stamatovic et al. [131] found increased vascular permeability and altered TJ organization with ageing in mice. Interestingly, they found that these changes were associated with decreased brain endothelial sirtuin-1 (Sirt-1) expression and that Sirt-1 regulates BBB function (e.g. cell-specific knockout increases permeability). Importantly, they also found evidence that down-regulation of brain endothelial Sirt-1 also occurs in the human brain with ageing.

In addition to long-term temporal changes in function of the blood–brain barriers, there is growing evidence for short-term changes not only in response to injury but also in the normal brain. Thus, the choroid plexus displays a circadian rhythm, with circadian changes in the expression of ‘clock genes’ [132]. Importantly, those changes may, via the CSF, impact the suprachiasmatic nucleus of the hypothalamus, the location of the main mammalian circadian clock [132]. One impact of the choroid plexus circadian rhythm are changes in the clearance of metabolic waste products [133]. A recent study on the drosophila ‘equivalent’ of the BBB also detected a circadian rhythm with alterations in efflux transport [20]. There has been considerable interest on the effects of sleep on the glymphatic system and metabolic clearance [134]. How these barrier and fluid flow changes are integrated merits further attention.

### Spatial heterogeneity

As well as temporal heterogeneity, there is growing evidence of spatial heterogeneity in the NVU/BBB. Thus, there is evidence of differences in barrier function between different areas of the brain that might impact the course of neuropathology [135]. Interestingly, Wang et al. recently discovered that the role of the Norrin and Wnt7a/Wnt7b signaling systems in NVU/BBB and blood-retinal barrier development shows marked regional heterogeneity [129].

In an important study, Vanlandewijck et al. [136] used single cell transcriptomics to examine changes in endothelial and mural cell gene expression going from arterial, capillary, to venous vessels (zonation). That study, and the associated searchable database, demonstrates marked effects of zonation on gene expression, e.g. for some genes there are marked changes in expression between arterial, capillary and venous endothelial cells. It should be noted that at the protein level differences in expression have be found even between adjacent endothelial cells (e.g. [137]).

## Future directions

Brain barrier and brain fluid research continues to be a vibrant field with many groups participating in this exciting area of CNS investigation. Insights from scientists in other areas, such as imaging, bioengineering and computer modeling, are helping to advance the field. Research has also been inspired by discoveries in other tissues. As with 2018, 2019 promises to be a year of provocative and important findings and we look forward to further excellent contributions to *Fluids Barriers of the CNS*.
